# „Agonist-antagonist myoneural interface (AMI)“: Innovative Versorgungsmöglichkeit für Beinamputierte?

**DOI:** 10.1007/s00113-025-01536-3

**Published:** 2025-03-06

**Authors:** M. N. Kalff, V. Hoursch, N. Kirsten, L. A. Pardo Jr., K. Kasprzak, M. Egger, S. N. Schmidt, S. Sehmisch, J. Ernst

**Affiliations:** 1 Medizinische Hochschule Hannover, Klinik für Unfallchirurgie, Carl-Neuberg-Straße 1, 30625 Hannover, Deutschland; 2 Universitätsmedizin Göttingen, Klinik für Unfallchirurgie, Orthopädie und Plastische Chirurgie, Göttingen, Deutschland

**Keywords:** Untere Extremität, Chirurgische Amputation, Myodese, Myoplastik, Propriozeption, Lower extremity, Surgical amputation, Myodesis, Myoplasty, Proprioception

## Abstract

Das „agonist-antagonist myoneural interface“ (AMI) ist ein innovativer Ansatz zur Rekonstruktion der Propriozeption und zur intuitiveren motorischen Kontrolle nach Gliedmaßenverlust. Es basiert auf der Nachbildung der natürlichen biomechanischen Beziehung zwischen Agonisten- und Antagonistenmuskeln, um dem Prothesennutzer eine bidirektionale Kommunikation zwischen der Prothese und seinem peripheren Nervensystem zu ermöglichen. Neurovaskulär gestielte Agonisten-Antagonisten-Muskelpaare werden durch eine adaptierte Sehnennaht in einem Gleitlager miteinander verbunden, sodass Spannungsänderungen während der Bewegung ein propriozeptives Feedback erzeugen. Dieses Feedback wird über afferente Nervenbahnen zum Zentralnervensystem weitergeleitet, wodurch eine Wahrnehmung der Gelenkposition des ursprünglich von dem Muskelpaar geführten Gelenks ermöglicht und gleichermaßen die Prothesensteuerung erleichtert wird. Das AMI scheint eine Integration der Prothese in die bestehenden neuronalen Netzwerke zu ermöglichen und verbessert sowohl die Steuerung der Prothese als auch die sensorische Diskriminierung. Im Vergleich zur Standardoperationstechnik (Myodese oder Myoplastik) mit einer nahezu rein mechanischen Transposition der residuellen Stumpfmuskeln reduziert AMI die kognitive Belastung während der Prothesennutzung und vermittelt ein natürlicheres Bewegungsgefühl, was das Embodiment positiv beeinflusst. Insgesamt markiert AMI einen bedeutenden Fortschritt in der Mensch-Maschine-Integration und stellt einen vielversprechenden Ansatz, um die Lebensqualität von Menschen mit einem Gliedmaßenverlust nachhaltig zu verbessern, dar.

## Abstract (en)

The agonist-antagonist myoneural interface (AMI) is an innovative approach to restoring proprioception and achieving more intuitive motor control following limb loss. This cutting-edge technique replicates the natural biomechanical relationship between agonist and antagonist muscles, enabling bidirectional communication between a prosthesis and the user’s peripheral nervous system. Through the transposition of neurovascularly pedicled agonist-antagonist muscle pairs, which are reconnected via an adapted tendon suture and positioned within a gliding mechanism, AMI generates proprioceptive feedback during movement. Changes in tension within these muscle pairs produce signals that are transmitted to the central nervous system via afferent nerve pathways, enabling users to perceive the joint position of the limb that was originally governed by the muscle pair. This enhanced sensory input significantly facilitates control of the prosthesis. The AMI appears to enable an integration of the prosthesis into the body’s existing neural networks and improve motor control of the prosthesis and the sensory discrimination. Compared to traditional surgical techniques (myodesis or myoplasty) with a purely mechanical transposition of residual stump muscles, AMI reduces the cognitive burden during the use of the prosthesis and delivers a more natural sense of movement, fostering a profound sense of embodiment. In summary, AMI represents a significant leap forward in human-machine integration. By enhancing both the functionality and user experience of prosthetic devices, it provides a very promising transformative solution for sustainable improvement of the quality of life for individuals living with limb loss.

Pro Jahr werden in Deutschland 62.000 Amputationen durchgeführt. Transtibiale Amputationen machen 10,4 % aller Amputationen aus, während transfemorale Amputationen weiterhin den größeren Anteil mit 14,5 % darstellen [1].

Ein Muskel ist ein spezialisiertes Gewebe, das für die Bewegung und Stabilität des Körpers verantwortlich ist. Muskeln bestehen aus kontraktilen Zellen, die sich kontrahieren und entspannen können, um Kraft zu erzeugen. Diese Muskeleigenschaften ermöglichen Bewegungen, die Funktion innerer Organe und die Aufrechterhaltung der Körperhaltung [2].

Adäquates Management der residualen Muskeln sichert die Funktionalität und den Komfort des Amputationsstumpfes

Residuale Muskeln spielen auch eine entscheidende Rolle im Amputationsstumpf, da sie sowohl für die Funktionalität als auch den Komfort des Stumpfes von großer Bedeutung sind. Eine funktionelle, gezielte Transposition der verbleibenden Stumpfmuskulatur ist Grundlage der erfolgreichen Prothesenversorgung [3, 4].

Anforderungen an die residuelle Muskulatur im Stumpf:

1. Kraftübertragung und Bewegungssteuerung:
  *Restfunktion und Prothesensteuerung*: Muskeln im Stumpf sind elementar, um Bewegungen effektiv auf die Prothese zu übertragen. Sie ermöglichen eine präzise Steuerung und triggern Bewegung und Funktion durch ihre Restfunktion oder elektrische Aktivität [4].
2. Schutz und Polsterung
  *Weichgewebepolster*: Muskeln bieten eine wichtige Polsterung des Knochens im Stumpf und verhindern, dass der Knochen direkten Druck auf die Haut ausübt, was zu Schmerzen oder Ulzerationen führen könnte [4].
  *Druckverteilung*: Muskeln unterstützen eine gleichmäßige Verteilung der Druckkräfte, die durch den Kontakt der Prothese mit dem Untergrund entstehen [4].
3. Propriozeption und Balance:
  *Körperwahrnehmung*: Verbleibende Muskeln im Stumpf tragen zur Propriozeption bei, was dem Nutzer hilft, die Position des Stumpfes und der Prothese im Raum besser wahrzunehmen [5].
  *Gleichgewicht*: Aktive Muskulatur unterstützt die Balance und beugt Stürzen vor, da sie die Lastverlagerung auf der Prothese erleichtert [4].

## Gegenwärtiges chirurgisches Muskelmanagement

### Myoplastik

**Abb. 1:**
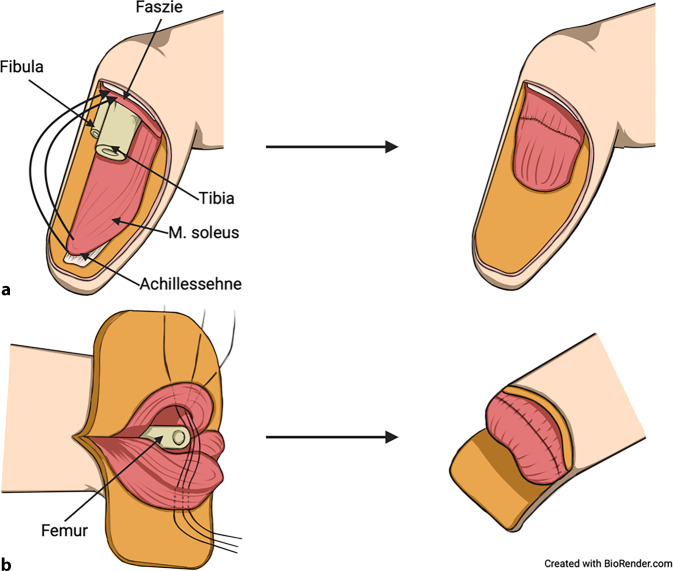
Schematische Darstellung der Myoplastik. **a** Unterschenkel: Die dorsale Stumpfmuskulatur (M. soleus und/oder Mm. gastrocnemius medialis et lateralis) wird über Nähte mit der ventralen proximalen Unterschenkelmuskulatur koaptiert. Dazu kann bzw. können die Muskelfaszie oder Teile des Muskel-Sehnen-Übergangs zusätzlich mit der ventralen Körperfaszie koaptiert werden, um die Myoplastik zu stabilisieren. **b** Oberschenkel: Die dorsalen und ventralen Muskellappen werden durch transmuskuläre Nähte um das distale Femur geschwungen. Dies kann durch die Koaptation der lateralen Abduktoren- und der medialen Adduktorenmuskelgruppe ergänzt werden. (Erstellt mit BioRender.com)

Bei dieser Technik werden Muskeln um das distale Knochenende durch eine Muskel-Muskel-Naht miteinander koaptiert (Abb. 1; [3, 4, 6]). Sofern möglich, sollten die oberflächlichen Muskelfaszien vom (resorbierbaren) Nahtmaterial gefasst werden, um Muskelischämien und ein Ausreißen der Nähte aus dem Muskelgewebe zu vermeiden. Die gebildete Muskelschlinge kann an benachbarten Faszien oder dem Periost fixiert werden. Ziel dieser Methode ist es, eine ortsstabile, polsternde Muskelmasse über dem knöchernen Amputationsstumpf zu schaffen, um gleichzeitig die Funktionalität, Stabilität, Belastbarkeit des Stumpfes zu optimieren und den Tragekomfort des Prothesenschafts zu erhöhten [3, 4, 6].

### Myodese

**Abb. 2:**
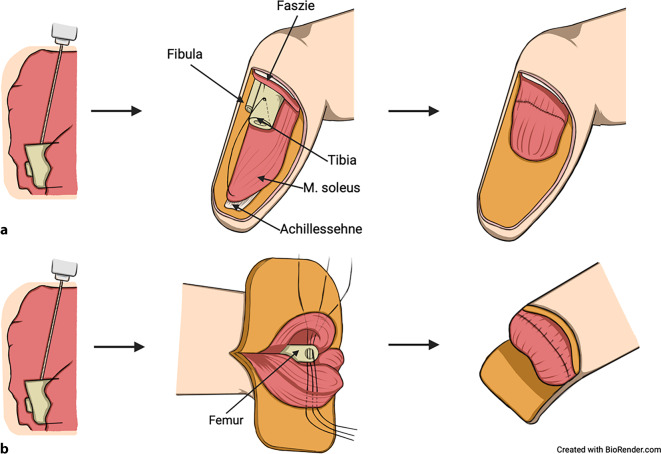
Schematische Darstellung der Myodese. **a** Unterschenkel: Platzierung von 2 monokortikalen Bohrlöchern (bei 3 und 9 Uhr) an der distalen, ventralen Tibia entsprechend den Grundprinzipien des Muskelmanagements (s. Text). Anschließend wird der Muskel-Sehnen-Anteil der dorsalen Unterschenkelmuskulatur über transossäre Nähte durch die Bohrlöcher geführt und am Periost und/oder mit der ventralen Streckmuskulatur verbunden und um die ventrale Tibia geschlungen. **b** Oberschenkel: Das Femur wird von ventral nach dorsal und von lateral nach medial durchbohrt. Anschließend werden der dorsale und ventrale Muskellappen sowie, wenn möglich, ein lateraler und medialer Muskellappen über transossäre Nähte um das Femur fixiert. (Erstellt mit BioRender.com)

**Abb. 3:**
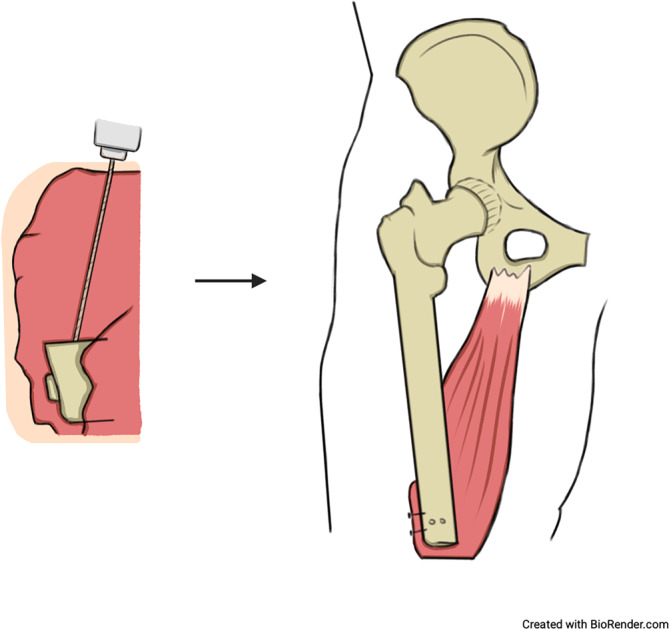
Schematische Darstellung der Adduktorenplastik nach Gottschalk [4, 9]. Es werden 2 asymmetrische Weichteillappen in der Sagittalebene angelegt. Der längere Lappen wird medial gebildet und umfasst den M. adductor magnus, während der kürzere, laterale Lappen aus der Quadrizepsmuskulatur gebildet wird. Die Osteotomie des Femurs erfolgt 12 cm proximal des Kniegelenkspalts. Anschließend wird die Sehne des M. adductor magnus nach lateral um den Knochen geschlagen und mit 2 Bohrlöchern am Femur fixiert. Abschließend wird der Quadrizeps über den distalen Femur geschlagen und dorsal fixiert, um die Stabilität und Funktionalität der Weichteildeckung zu gewährleisten. (Erstellt mit BioRender.com)

Die Myodese wiederum fixiert Muskeln zusätzlich mithilfe einer transossären Naht durch Bohrlöcher am Knochen – alternativ wurden Knochenanker beschrieben (Abb. 2; [3, 7, 8]). Bei der Adduktorenplastik nach Gottschalk für die transfemorale Amputation wird ein medialer Lappen aus dem M. adductor magnus am sehnigen Anteil über zwei 2‑mm-Bohrlöcher mit nichtresorbierbaren Nähten am Femur in 5°- bis 10°-Adduktion fixiert (Abb. 3). Der laterale Anteil der Quadrizepsmuskulatur wird in gleicher Weise dorsal in Hüftstreckung fixiert. Die Fascia lata wird mit der medialen Faszie vernäht, oft ergänzt durch transossäre Fixierungen zur Stabilisierung [9].

Es gelten chirurgische Grundprinzipien wie der maximale Erhalt funktioneller Muskelmasse sowie stabile und funktionelle Refixation unter Erhalt des gesamten Bewegungsradius zur Vermeidung von Muskelkontrakturen bei gleichzeitigem Erhalt der notwendigen der physiologischen Vorspannung [4, 9]. Um ein belastungsstabiles Einheilen der Muskelnähte zu gewährleisten, wird eine Ruhigstellung von 3 bis 6 Wochen empfohlen. Die gezielte Rehabilitation mit Muskelaufbau und -kräftigung ist ein zentrales Element der Rehabilitation nach einer Amputation. Durch gezielte Übungen werden Kraft, Flexibilität und Kontrolle der verbleibenden Muskulatur verbessert, um die Prothesenanpassung und ihre Nutzung zu erleichtern [4].

Die oben genannten Maßnahmen sind entscheidend für die optimale Funktionalität der Stumpfmuskulatur.

### Grenzen und Komplikationen

Zu den häufigen Komplikationen gehört die Dislokation oder das Ausreißen von Myodese- und Myoplastiknähten, was mit einer unzureichenden Weichteildeckung durch lediglich Vollhaut einhergehen kann. Weitere Folgen sind Stumpfatrophie, die Entstehung von Druckstellen und Ulzerationen sowie eine erhöhte Anfälligkeit für die Entwicklung von Stumpfschmerzen. Um eine stabile Vernarbung der Muskelnaht zu gewährleisten, hat Brückner eine Immobilisation des Kniegelenks in Streckung bis zur vollständigen Wundheilung empfohlen [4]. Eine nachträgliche Dislokation der Muskeln vom Stumpfende, die in etwa 6 % der Fälle eine chirurgische Revision erforderlich macht, tritt insbesondere dann auf, wenn der Stumpf an eine Prothese mit einem zu engen Schafteingang angepasst wird [10].

## Innovativer Ansatz zur Rekonstruktion der Propriozeption und zur intuitiveren motorischen Kontrolle

### Prinzip

Der Amputationschirurg Ernest Martin Burgess berichtete nach seiner Rückkehr von einem Besuch bei seinem Kollegen Marian Weiss in Polen von einer effektiven Stumpfpolsterung und Wiederherstellung der Propriozeption, die er dort bei den amputierten Patienten beobachtet hatte [11]. Diese klinischen Beobachtungen konnten jedoch weder 1967 noch bis heute für die Myoplastik oder die Myodese objektiv nachgewiesen werden [12].

Die Propriozeption, auch als Lagesinn bezeichnet, beschreibt die Fähigkeit, die Position von Körperteilen im Raum selbst bei geschlossenen Augen präzise wahrzunehmen [5]. Dieser Sinn wird im Großhirn durch die Integration neuraler Informationen aus dem Kleinhirn, den Gehörgängen, der spinalen Ebene sowie aus peripheren Sinnesorganen in Muskeln, Sehnen, Gelenkkapseln und der Haut verarbeitet [13, 14]. Die peripheren Sinnesorgane spielen eine zentrale Rolle, wobei die Mechanorezeptoren, insbesondere die Muskelspindeln und Golgi-Sehnenorgane am Übergang zwischen Muskel und Sehne, die Hauptakteure sind. Diese Rezeptoren registrieren Veränderungen in Muskellänge, Bewegungsgeschwindigkeit und Spannung [15].

In den Extremitäten arbeiten Muskelpaare, die ein Gelenk überspannen, in einer funktionellen Einheit als Agonisten und Antagonisten zusammen [16]. Die afferente Signalübertragung von Mechanorezeptoren während der Bewegung der Gliedmaßen wird durch die An- und Entspannung bzw. Dehnung dieser Muskelgruppen codiert. Bei der Fußhebung beispielsweise bewirkt die Kontraktion der Fußheber (Extensoren und M. tibialis anterior) eine gleichzeitige Entspannung bzw. Dehnung der Fußsenker (Flexoren, einschließlich Mm. gastrocnemius, medialis, lateralis und soleus). Das derzeitige Vorgehen und die Anforderungen an die polsternde Funktion der Stumpfmuskulatur bei einer transtibialen Amputation berücksichtigen die anatomische und neuromechanische Kopplung von Agonisten und Antagonisten nur unzureichend. Schnelle Reaktionen zur Haltungskorrektur und zur Gleichgewichtskontrolle während des Gehens sind bei Beinamputierten aufgrund der gestörten motorischen Kontrolle und eingeschränkten propriozeptiven Wahrnehmung erheblich beeinträchtigt [17, 18].

**Abb. 4:**
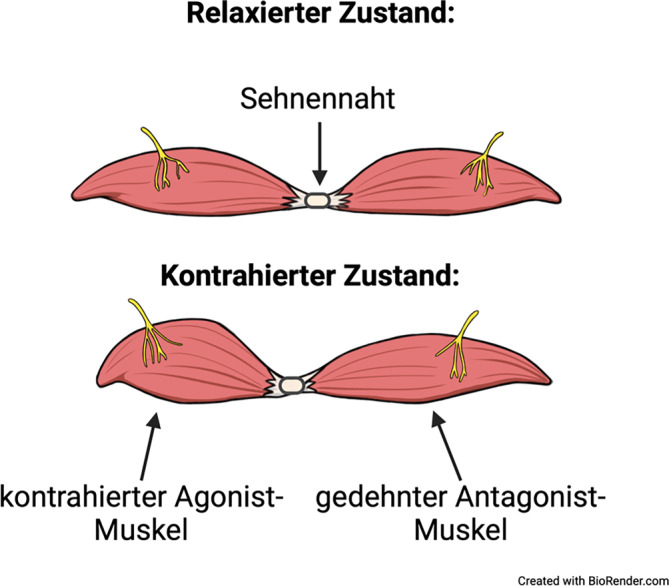
Schematische Darstellung des Grundkonzepts der Agonist-antagonist-myoneural-interface-Methode. Das Agonist-Antagonist-Muskelpaar wird über eine modifizierte Sehnennaht miteinander verbunden. Durch die neuromechanische Kopplung führt eine Kontraktion des Agonisten zu einer Dehnung des Antagonisten. Über diesen Mechanismus wird u. a. die Propriozeption wiederhergestellt. (Erstellt mit BioRender.com)

Die Grundidee des AMI-Verfahrens ist die Rekonstruktion der neuromechanischen Kopplung von zusammengehörigen Agonist-Antagonist-Muskelpaaren im Amputationsstumpf (Abb. 4). Jedes AMI-Konstrukt besteht aus 2 biomechanisch gekoppelten, funktionell gegensätzlich arbeitenden Muskeleinheiten (z. B. dem M. tibialis anterior und dem M. gastrocnemius) sowie ihren zugehörigen neuronalen Komponenten.

### Operatives Vorgehen

#### Transtibiale Amputation mit Bildung von 2 Agonist-antagonist myoneural interfaces

Ziel ist die Wiederherstellung der muskulären Kopplung von Fußhebung und -senkung (Agonist und Antagonist) zur Rekonstruktion der Ansteuerung des oberen Sprunggelenks (OSG) sowie der Inversion und Eversion zur Rekonstruktion der Ansteuerung des unteren Sprunggelenks (USG; [19]). Alle übrigen nichtgenutzten Muskeln des Fußes und Unterschenkels werden weit proximal abgesetzt und reseziert. Die Fibula wird mindestens 1–2 cm proximal der Tibia osteotomiert und das ventrale Drittel der Tibia angeschrägt [4, 6]. Die Präparation erfolgt unter anliegender Oberschenkelblutsperre.

Hautschnitt und Osteotomie werden je nach verbleibendem Haut-Weichteil-Mantel geplant. Der Hautschnitt erfolgt zunächst bis auf die ventrale und dorsale Faszie entlang der Einzeichnung. Es folgt ventral die Inzision der selbigen, sodass die oben genannte Muskulatur unbeschadet präpariert und entlang der Sehnen am ventrolateralen distalen Unterschenkel dargestellt werden kann. Die AMI-Muskelpaare werden proximal des Retinaculums und Malleolus lateralis abgesetzt und markiert. Nach der Osteotomie und Absetzung des Fußes unter Erhalt eines langen dorsalen Lappens erfolgen die Präparationen des M. tibialis posterior, des M. soleus und eines weiteren Bauches des M. gastrocnemius, der an seinem sehnigen Anteil von der Achillessehne vom M. soleus getrennt und entlang der Raphe unter Schonung des N. suralis vom benachbarten Bauch separiert wird. Die dorsalen Muskelpaare werden ebenso markiert. Die verbleibenden tiefen Flexoren, Peronäalmuskeln und Extensoren können proximal der Osteotomie reseziert werden.

Die durchtrennten Nerven werden danach zur Vermeidung von schmerzhaften Stumpfneuromen mit gezielten Techniken weiterbehandelt. Dazu zählen der selektive Nerventransfer auf sensible Hautnerven (Targeted Sensory Reinnervation, TSR, [20]), die Reinnervation motorischer Empfängernerven von Nicht-AMI-Muskeln (Targeted Muscle Reinnervation, TMR, [21, 22]) oder die Einbettung in ein kleines avaskuläres Muskeltransplantat (Regenerative Peripheral Nerve Interface, RPNI, [23, 24]). Diese Ansätze haben sich als wirksam erwiesen, um die Häufigkeit von Neurom- und Phantomschmerzen zu reduzieren [25].

Das Ziel der AMI-Methode ist die Wiederherstellung der Propriozeption

**Abb. 5:**
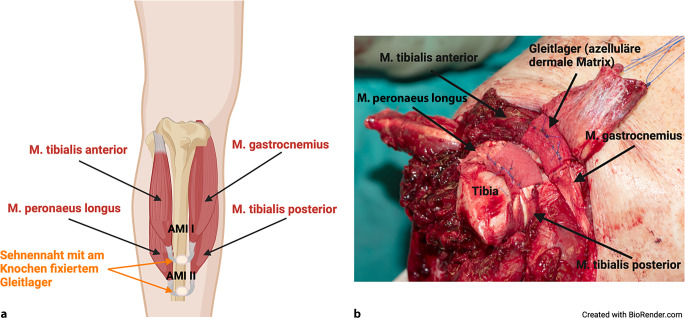
**a** Schematische Darstellung der Agonist-antagonist-myoneural-interface(*AMI*)-Konfigurationen bei transtibialer Amputation, **b** intraoperative Aufnahme einer transtibialen AMI-Amputation. (Erstellt mit BioRender.com)

Die dargestellten Muskelpaare werden durch eine modifizierte Sehnennaht am intramuskulären Anteil der Sehnen ventral der Tibia unter Vorspannung miteinander verbunden. Das AMI I bilden der M. tibialis anterior und ein Muskelbauch des M. gastrocnemius zur Rekonstruktion der OSG-Funktion; wenige Zentimeter distal vom ersten AMI wird der AMI II durch den M. peronaeus longus und den M. tibialis posterior in ähnlicher Weise zur Rekonstruktion der USG-Funktion gebildet (Abb. 5; [19]). In der Tiefe wird eine Redon-Drainage eingelegt und nach lateral ausgeleitet.

Der verbleibende M. soleus wird, sofern eine ausreichende Durchblutung gewährleistet ist, zur Stumpfdeckung von dorsal nach ventral über die AMI-Konstrukte I und II geschlagen. Anschließend wird der Muskel samt seiner Faszie an der ventralen oberflächlichen Körperfaszie fixiert. Nachfolgend werden der lange dorsale Hautlappen umgeschlagen und die Haut verschlossen [19].

Das Bein wird anschließend für 6 Wochen mit einer Knieimmobilisationsschiene in Kniestreckung fixiert, um Belastungen der noch unreifen, instabilen Sehnennaht zu vermeiden.

#### Transfemorale Amputation mit Bildung von 4 Agonist-antagonist myoneural interfaces

Das Grundprinzip des AMI bei der transfemoralen Amputation oder Knieexartikulation entspricht dem der transtibialen Amputation (s. oben). Die Osteotomie des Femurs wird etwa 12 cm proximal der Femurkondylen durchgeführt, um eine Überlänge zu vermeiden, insbesondere in Bezug auf die Aufbauhöhe des Knies.

**Abb. 6:**
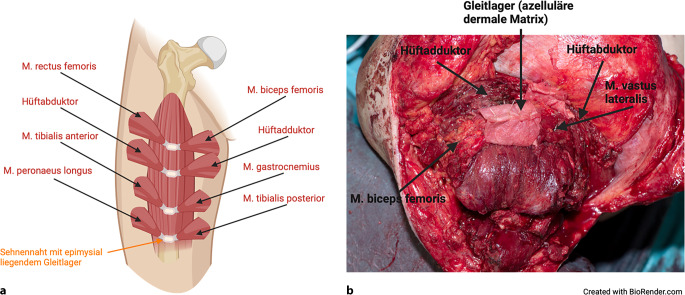
**a** Schematische Darstellung der Agonist-antagonist-myoneural-interface(AMI)-Konfiguration bei transfemoraler Amputation mit bis zu 4 AMI, **b** intraoperative Aufnahme einer transfemoralen AMI-Amputation mit 2 AMI. (Erstellt mit BioRender.com)

Je nach Indikation und verbleibenden Muskeln distal des Knies können idealerweise 4 AMI konstruiert werden (Abb. 6). Sofern möglich, werden diese aus 4 bis 8 neurovaskulär gestielten Muskellappen gebildet. Das AMI I dient der Ansteuerung des Kniegelenks und AMI II der Femurabduktion/-adduktion. Äquivalent zu der Muskeltransposition der transtibialen AMI rekonstruieren das AMI III das OSG und das AMI IV das USG [26]. Für die Kniegelenkfunktion wird der M. rectus femoris (Kniestreckung) mit dem medialen M. biceps femoris (Kniebeugung) verbunden. Das AMI II besteht aus einem Femurabduktor und -adduktor. Die Gleitlager der AMI-Muskeln liegen epimysial und werden nicht mithilfe von Knochenankern am Femur fixiert.

Die Muskelpaare werden durch eine Sehnennaht am intramuskulären Sehnenanteil unter Vorspannung verbunden

Falls die originären Muskeln für die Sprunggelenk-AMI nicht verfügbar sind, können regenerative AMI geschaffen werden. Hierzu werden die Nerven, die die entsprechenden Muskeln innervieren, mit kleinen avaskulären Muskeltransplantaten kombiniert, um diese zu reinnervieren und als Agonist-Antagonist-Paare zu koaptieren [27]. Zur Neuromprophylaxe können auch hier verschiedene selektive Nerventransfers erfolgen (s. oben; [20–25]).

## Diskussion

Die Erstbeschreiber der AMI-Methode konnten funktionelle und sensorische Vorteile der AMI, insbesondere die verbesserte motorische Kontrolle der Prothese und die Wiederherstellung der propriozeptiven Wahrnehmung, im Vergleich zu anderen Amputationstechniken aufzeigen [26, 28–30]. Durch die Wiederherstellung der Propriozeption können die Patienten die AMI-Muskeln gezielter ansteuern und die genaue Position des Phantomfußes im Raum wahrnehmen. Chiao et al. haben 5 verschiedene Patient Reported Outcome Measures (PROM) bei 31 Patienten mit einer transtibialen oder transfemoralen AMI-Amputation über einen Beobachtungszeitraum von 12 Monaten erhoben [28]. In allen 5 Fragebogen konnten sie eine signifikante Verbesserung der Lebensqualität im Vergleich zu der Zeit vor der Amputation zeigen. Im nächsten Schritt sollen AMI-Patienten mit Patienten, die eine Standardamputation erhalten haben, verglichen werden.

Klinische und funktionelle Tests an transtibialen und transfemoralen AMI-Patienten haben gezeigt, dass AMI zu einer langfristig stabileren Stumpfform und geringeren Umfangsschwankungen führen [26, 29]. Zudem ermöglichen sie ein präziseres Ansteuern der AMI-Muskeln sowie eine Reduktion zusätzlicher antagonistischer Kontraktionen und Kokontraktionen. Es berichteten 71 % der Patienten von einer verstärkten Phantomempfindung und verbesserten Propriozeption durch die Aktivierung der AMI-Muskeln. Grad und Ort der wahrgenommenen Phantombewegung korrelierten direkt mit der Spezifität und Intensität der AMI-Aktivierung [26, 29].

Von verbesserter Propriozeption durch die Aktivierung der AMI-Muskeln berichteten 71 % der Patienten

Die Signale der AMI-Muskeln sollen künftig über transkutane EMG-Elektroden abgeleitet werden, um ein mechatronisches, myoelektrisch gesteuertes aktives Sprunggelenk gezielt und willkürlich steuern zu können.

Die Arbeitsgruppe des Erstbeschreibers konnte erst kürzlich zeigen, dass die Kombination der AMI-Methode zur Steuerung eines mechatronischen, myoelektrisch geführten aktiven Sprunggelenks ein natürlicheres Gangbild nach einer Amputation ermöglicht sowie die Lebensqualität von Amputierten durch eine effizientere und natürlichere Bewegungsfolge im Vergleich zur Standardamputation verbessert [30]. Für die Studie wurden 7 Patienten mit einer transtibialen AMI-Amputation mit 7 Patienten mit einer transtibialen Standardamputation verglichen. In der AMI-Gruppe konnten die Fußhebung und -senkung während des Gehens aktiv über transkutane EMG-Elektroden im Prothesenschaft gesteuert werden. Alle Probanden hatten zuvor marktverfügbare passive (carbonfaserbasierte) Prothesenfüße genutzt.

Zur Objektivierung der afferenten Muskelsignale aus den AMI-Muskeln wurden Muskelfaszikeldehnungen („muscle fascicle strains“) mithilfe von Ultraschall und EMG-Signalen analysiert. Die Ergebnisse zeigten, dass die AMI-Gruppe 18 % der physiologischen afferenten Impulsrate wiederherstellen konnte, während in der Kontrollgruppe keine afferenten Muskelsignale nachweisbar waren [30].

Diese 18 %ige „afferente Augmentation“ der AMI-Gruppe führt beim Gehen auf der Ebene mit verschiedenen Gehgeschwindigkeiten sowie bei 5 %iger Steigung bzw. Gefälle, Auf- und Abgehen von Treppen und Überqueren von statischen Hindernissen zu einer überlegeneren Performance im Vergleich zu den Probanden, die eine Standardamputation erhielten. Die AMI-Gruppe erreichte zudem eine höhere kinematische Symmetrie zur gesunden Gegenseite und war in der Lage, ebenerdige Strecken (+41 %), Gefälle (+32 % bis +39 %) und Treppen (+41 % bis +43 %) schneller zu überwinden. Diese Gehgeschwindigkeiten waren vergleichbar mit den Normdaten gesunder Personen [30]. Beim Überqueren statischer Hindernisse konnten die AMI-Probanden diese ohne ein Abstoppen schneller bewältigen, indem sie das Sprunggelenk in Dorsalextension positionierten und sich nach dem Aufsetzen des Prothesenfußes aktiv und zügig nach vorn abdrückten. Dadurch wurde ein flüssiger und ungestörter Gang ermöglicht [30].

Die Kombination von AMI mit einem myoelektrisch-gesteuerten, aktiven Sprunggelenk ermöglicht ein physiologischeres Gangbild

Zusammengefasst zeigten die in allen 3 Szenarien analysierten biomechanischen, kinetischen und kinematischen Parameter ein nahezu physiologisches Reaktionsverhalten, das eine deutlich größere Ähnlichkeit zur intakten Gliedmaße aufwies und sich beträchtlich von den Ergebnissen bei der Nutzung passiver Prothesenfüße unterschied.

Die ersten Ergebnisse der AMI-Methode sind vielversprechend. Dennoch bleibt zu reevaluieren, ob der operative und zeitliche Mehraufwand gegenüber den funktionellen Gewinnen gerechtfertigt ist, und für welche Patientengruppen die AMI-Methode indiziert ist. Zudem muss überprüft werden, ob die Methode auch bei Revisionen, wie z. B. nach ausgerissener Myoplastik oder Myodese sowie bei insuffizienter Weichteildeckung anwendbar ist.

## Resümee

An der Medizinischen Hochschule Hannover versorgen wir seit Januar 2022 Patienten mit der vorgestellten AMI-Methode und können ihren positiven Einfluss in den mehr als 50 Versorgungen in unserer klinischen Nachsorge und in stattfindenden wissenschaftlichen Nachuntersuchungen vergleichbar beobachten. Das AMI erweist sich als vielversprechender Ansatz zur Verbesserung von Kontrolle, Funktionalität, Komfort und dem allgemeinen Benutzererlebnis, wodurch es eine bedeutende Innovation im Bereich der Neuroprothetik und Rehabilitation darstellt.

### Infobox Weiterführende Informationen

https://www.media.mit.edu/projects/agonist-antagonist-myoneural-interface-ami/overview/

https://www.media.mit.edu/projects/agonist-antagonist-myoneural-interface-ami/frequently-asked-questions/

https://www.mhh.de/unfallchirurgie/leistungsspektrum/innovative-amputationsmedizin

https://nife-hannover.de/ags/innovative-amputationsmedizin/

## Fazit für die Praxis

- Das AMI ist eine innovative mechanoneurale Mensch-Maschine-Schnittstelle. Die bisherigen Ergebnisse zeigen, dass die AMI-Methode durch die Wiederherstellung afferenter Signale und in Kombination mit einer bionischen Prothese eine biomechanische Integration ermöglicht. Dies führt nicht nur zu einer signifikanten Funktionsverbesserung, sondern auch zu einem physiologischeren Gangbild im Vergleich zu Standardamputationen. Die zusammengefassten Vorteile der AMI-Methode sind: verbesserte Prothesenkontrolle, erweiterter nutzbarer Funktionsbereich der Prothesenversorgung, verringerte Muskelermüdung und -atrophie. Das natürliche Bewegungsmuster wird wiederhergestellt; Rückmeldemechanismen, insbesondere die Propriozeption, werden rekonstruiert und die Neuroplastizität gefördert. Prothesenakzeptanz, Embodiment, Ownership sowie Sense of Agency der Beinamputierten werden gesteigert.
